# Breast cancer screening and diagnosis in older adults women in Brazil: why it is time to reconsider the recommendations

**DOI:** 10.3389/fpubh.2023.1232668

**Published:** 2023-08-02

**Authors:** Aline Ferreira Bandeira Melo Rocha, Ruffo Freitas-Junior, Leonardo Ribeiro Soares, Glalber Luiz Rocha Ferreira

**Affiliations:** ^1^Faculty of Medicine, Federal University of Goiás, Goiânia, Goiás, Brazil; ^2^Advanced Center for Breast Diagnosis (CORA), Federal University of Goiás, Goiânia, Goiás, Brazil; ^3^Goiás State Education Department, Goiânia, Goiás, Brazil

**Keywords:** breast cancer, breast cancer screening, clinical staging of breast cancer, mammography screening, older adults women, Brazil

## Abstract

**Introduction:**

Breast cancer screening in women of 70 years of age or older remains controversial due to a lack of studies that include women of this age.

**Methods:**

This ecological study evaluated data from the Brazilian National Health Service (SUS) on breast cancer screening and staging in this age group compared to 50–69-year olds, for Brazil as a whole and for its geographical regions, between 2013 and 2019. A secondary database was obtained from the outpatient data system of the SUS’s Informatics Department, the Brazil Oncology Panel, the Brazilian Institute of Geography and Statistics, the Supplementary Health Agency and the Online Mortality Atlas.

**Results:**

There was a marked reduction in screening in women ≥70 years of age (annual percent change [APC] –3.5; *p* < 0.001) compared to those of 50–69 years of age (APC-2.2; *p* = 0.010). There was a trend towards an increase in clinical staging, with a greater occurrence of stages III and IV in the ≥70 group (44.3%) compared to the women of 50–69 years of age (40.8%; *p* < 0.001).

**Conclusion:**

Considering the increasing age of the Brazilian population and the heterogeneity among older adults women, screening for the over-70s within the SUS merits greater debate insofar as the implementation of public policies is concerned.

## 1. Introduction

Breast cancer is the most common form of cancer worldwide, with 2.3 million new cases in 2020 according to global statistics (1). This important public health issue for many countries is the fifth cause of death from cancer in general and the most common cause of cancer-related death in women (1). In Brazil, excluding non-melanoma skin cancer, breast cancer is the most common form of cancer (2, 3), with an estimated 73,610 new cases in 2023 (3). Moreover, breast cancer is the principal cause of death from cancer in the female population within the country (2).

The condition is considered one of the current challenges of an aging population, since longevity is the principal risk factor for cancer, with a substantial rise in the number of cases and related mortality as age increases (3–5). The incidence of breast cancer continues to rise up to 75–79 years of age and 26% of the annual deaths from breast cancer are attributed to diagnosis after 74 years of age (6, 7).

Despite the increase in the incidence of breast cancer with aging, the screening of women ≥70 years of age remains controversial, since no prospective, controlled and randomized studies have been conducted with women in this age group. Consequently, most of the data involving breast cancer screening in this population is obtained by extrapolating results from other age groups (5, 7–9).

Observational studies and prediction models offer the best currently available data on the risks and benefits of breast cancer screening in this age group. The reported screening benefits include a reduction in mortality (5, 6, 10), an increase in the diagnosis of early stage tumors, which require less invasive treatment (8), greater sensitivity and specificity of mammograms (6, 7, 11, 12), a low rate of false-positive mammograms and biopsies (5, 13), and a minimal rate of over-diagnosis compared to younger populations (14).

Notwithstanding, there is substantial heterogeneity with respect to the comorbidities and life expectancy of older women, and controversy remains with regards to the implementation of breast cancer screening for the female population ≥ 70 years of age. Most screening programs worldwide offer mammograms to women from 40 to 50 years of age until 69–74 years of age (15) irrespective of the physical status of these women.

Practice guidelines have recommended breast cancer screening in older women unless the woman’s comorbidities limit her life expectancy (6, 16–18). Metrics on the performance of screening are increasingly favorable for women of 75–90 years of age, with no evidence supporting the interruption of screening based on chronological age alone but, rather, incorporating decisions based on the patient’s individual preferences, on comorbidities and on the state of health of these older adults women (11).

In Brazil, the Ministry of Health guidelines for the early detection of breast cancer discourage routine mammograms for women ≥70 years of age (19). Conversely, the Brazilian Society of Mastology, the Brazilian College of Radiology and Diagnostic Imaging and the Brazilian Federation of Gynecology and Obstetrics Societies recommend annual mammogram screening for women of 40–74 years of age. Screening is also recommended for the group of women of 75 years of age or older with a life expectancy of at least seven remaining years (7).

Few studies have reported on clinical staging at the time of diagnosis in populations of ≥70 years of age. While some studies have shown stages I and II to be more common (20–22), others have reported a reduction in the incidence of *in situ* disease and stage I (23), an increase in the prevalence of stage IV (24), and larger tumors and nodal involvement at presentation (25). Nevertheless, such differences can be partially explained by differences in national screening programs for this age group.

The aging of the Brazilian population implies a possible increase in the cases of breast cancer in women ≥70 years of age with a heterogenous state of health. Concomitantly, there is a lack of studies on this subject conducted in low-and-middle income countries (LMIC) in general. The current study was designed to evaluate Brazilian data on breast cancer screening and staging in the female population ≥ 70 years of age. Our hypothesis is that mammographic screening in the older adults population has decreased over time, contributing to the late diagnosis of breast cancer in this population.

## 2. Materials and methods

This ecological study involving a time-trend analysis evaluated breast cancer screening coverage and clinical staging using data from the Brazilian National Health Service (SUS) in women ≥70 years of age in Brazil, both in the country as a whole and in its five geographical regions (North, Northeast, Southeast, South and Midwest), between 2013 and 2019. Data from the group of women of 50–69 years of age, which is the age group included in the Ministry of Health recommendations for breast cancer screening within the National Health Service, were used for the purpose of comparison.

Due to the fact that the database was collected from different sources with different forms of age classification, and to allow statistical comparison between groups of women (screening recommended and not recommended by the Ministry of Health), only two age groups were used (50–69 versus ≥70 years).

A secondary database was created by extracting outpatient data from the SUS’s Informatics Department (SIA/DATASUS) regarding the number of mammograms performed (26) and from the Brazil Oncology Panel of the SUS Informatics Department (DATASUS) with respect to data on clinical staging (27). Furthermore, the Brazilian Institute of Geography and Statistics (IBGE) provided data regarding the target population in the country (28) and the female population covered by supplementary healthcare was calculated according to data from the Supplementary Health Agency (ANS) (29). Finally, data on mortality were collected from the Online Cancer Mortality Atlas (30).

The internal review board of the Federal University of Goiás Teaching Hospital approved the study protocol under reference CAAE: 56747022.0.0000.5078. Since the study used freely accessible and unrestricted secondary data, the requirement for informed consent was waived.

### 2.1. Target population

Given that the last census carried out in Brazil was in 2010, population projections were used to calculate the target population for the period between 2013 and 2019. The data were extracted from the IBGE’s *Projections for the population of Brazil and its states by sex and age: 2010–2060*, updated in 2018 (28). Based on this population projection, the percentage of women who had healthcare insurance was then obtained from ANS for each year of the study period (29), to calculate the estimate of the procedures.

### 2.2. Breast cancer screening coverage

Although the Ministry of Health does not recommend breast cancer screening for women ≥70 years of age, with the aim of obtaining similar data to compare with the data for the group of women of 50 to 69 years of age it was decided to use the same means of calculating coverage for both groups.

The number of mammograms performed annually between 2013 and 2019 was established according to two procedure codes recorded in the SIA/DATASUS outpatient data system: bilateral screening mammography (02.04.03.018–8) and mammography (02.04.03.003–6) (26). The analysis was restricted to the period beginning in 2013 since the Oncology Panel data referring to clinical staging are only available from that year onwards. In relation to the SUS data on breast cancer screening, under-notification is unlikely, since healthcare establishments need to register all mammograms performed in order to receive payment for the services provided.

To reach a more realistic estimate of procedures, the screening coverage goal, for each locality and period, was calculated by subtracting the percentage of women with supplementary healthcare, so that the procedures offered within the public healthcare system would not be overestimated (31).

Based on the assumption that screening would be performed every 2 years for 100% of the target population, the expected number of exams per year for the target population was calculated so that the annual need for screening mammograms corresponds to half of the female population in analysis (31).

Estimated coverage was expressed as a percentage and calculated from the ratio of the number of exams performed and the number of expected exams in the target population (31, 32). The years of 2020 and 2021 were excluded to avoid a bias caused by the effect of the COVID-19 pandemic on breast cancer screening.

### 2.3. Clinical staging

Data on clinical staging, available for 2013 onwards, were filtered to include stages I, II, III, and IV alone as these are the stages registered at chemotherapy, radiotherapy or both in the Oncology Panel platform (27). These data were then divided into two groups, the first consisting of stages I and II and representing the initial phases of breast cancer, and the second consisting of stages III and IV and representing advanced stages of the disease. Stage 0 was excluded since it represents *in situ* ductal carcinoma, a pre-cancerous lesion not considered to be breast cancer.

### 2.4. Statistical analysis

Trends in breast cancer screening rates were analyzed by evaluating the annual percent change (APC) in the estimated breast cancer screening coverage for Brazil as a whole and for its geographical macro-regions. Poisson regression models were applied for these calculations, using the JoinPoint Regression software program, version 4.9.0.1 of February 2022 (33). The 95% confidence intervals (95%CI) were calculated and value of *p*s <0.05 were considered statistically significant.

In the analysis and interpretation of the results, estimated breast cancer screening coverage was considered to have increased when there was a rising trend and when the minimum value of the confidence interval (CI) was above zero. It was considered to have fallen when there was a negative trend in the APC and the maximum value of the CI was less than zero. Coverage was deemed to have remained stable when, irrespective of the value of coverage, the minimum CI value was less than zero and the maximum was above zero.

Clinical staging for the whole country and for the different regions was characterized using absolute and relative frequencies. The comparison of staging between the ≥70 years of age group and the group of 50–69 years of age was performed using Pearson’s chi-square test. Poisson regression analysis was used to analyze trends in staging between 2013 and 2019. Breast cancer screening coverage rates were compared between the groups using Student’s t-test. The data were analyzed using the Statistical Package for the Social Sciences (SPSS), version 26.0 (34). The significance level adopted was 5% (*p* < 0.05).

## 3. Results

In 2013, the female population of Brazil ≥70 years of age consisted of 5,978,034 women, a number that had increased to 7,515,477 by 2019, representing a rise of 25.7% in this population over that timespan (28). The number of mammograms approved for payment in 2013 and 2019 was 296,116 and 274,957, respectively, at a total annual cost of R$ 12,452,213.50 and R$ 11,390,947.23, respectively (26). This represents a reduction of 7.14% in the exams paid for within the National Health Service over the period.

Over the study period, 35,099 deaths from breast cancer occurred in the over-70s group, with 4,1,179 occurring in 2013 and 5,857 in 2019, representing an increase of 40.15% (30). For the 50–69 years group, 51,654 deaths were registered over the period, with 6,565 in 2013 and 8,291 in 2019, representing an increase of 26.29% (30). The adjusted mortality rate increased over the period, as shown in Table 1, with this increase being proportionally greater for the over-70s group compared to the 50–69-year old group (30).

**Table 1:** Mortality rates from breast cancer per 100,000 women in Brazil between 2013 and 2019, adjusted for the world population, according to age group.

Age group	2013	2014	2015	2016	2017	2018	2019
≥70 years	66.35	66.81	70.98	69.83	71.71	75.26	73.37
50–69 years	37.56	37.17	38.06	38.32	38.95	38.80	39.64

When stratified according to the geographic region of the country, coverage was greater in the Southeast and South for both groups over the years analyzed (Table 2); however, breast screening coverage for the group of women ≥70 years of age was significantly lower compared to the 50–69 years age group. Table 2 shows the annual coverage for Brazil and its geographical regions for the study period.

**Table 2:** Comparison of percentage breast cancer screening coverage within the public healthcare system between women ≥70 years of age and women of 50–69 years of age, between 2013 and 2019 in Brazil and its geographical regions.

Coverage (%)/Regions	2013		2014		2015		2016		2017		2018		2019	
	≥70	50–69	≥70	50–69	≥70	50–69	≥70	50–69	≥70	50–69	≥70	50–69	≥70	50–69
Brazil	13.3	37.3	12.9	38.8	12.2	37.5	12.0	38.1	11.3	37.6	11.1	34.8	10.8	33.9
Midwest	8.1	20.6	7.5	20.4	5.5	15.4	5.9	17.1	5.3	16.0	5.4	15.4	5.8	18.0
Northeast	7.8	27.7	6.9	29.1	7.0	29.4	7.1	32.0	6.2	32.4	5.7	26.4	5.7	25.6
North	4.8	15.0	5.6	18.0	5.1	15.7	4.3	13.4	4.8	15.2	3.9	12.9	3.9	12.7
Southeast	18.9	47.4	19.3	51.3	18.0	49.5	17.4	48.6	16.4	47.1	16.4	45.5	15.9	43.8
South	15.8	44.9	14.4	42.4	14.2	41.7	14.3	42.9	14.4	42.4	14.1	41.5	13.8	40.2

**Figure 1:**
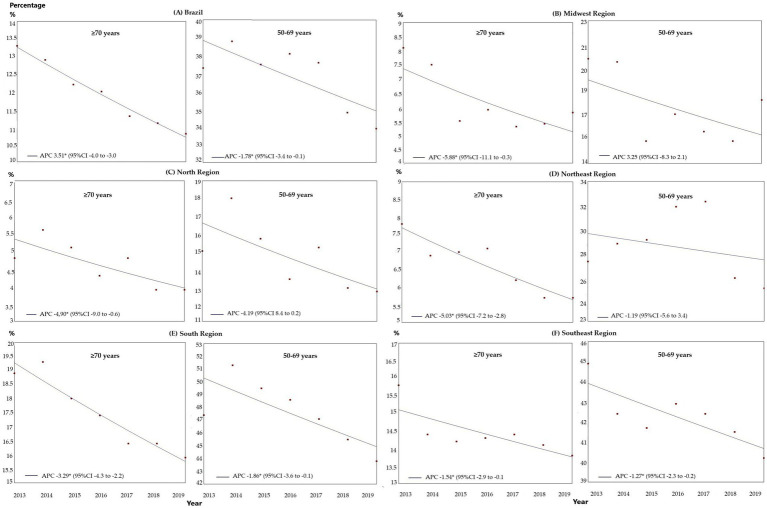
Comparison of the trends in breast cancer screening in Brazil within the public healthcare system between women ≥70 years of age and women of 50–69 years of age, between 2013 and 2019, in Brazil and the geographical regions: **(A)** Brazil; **(B)** Midwest; **(C)** North; **(D)** Northeast; **(E)** Southeast; and **(F)** South. APC, annual percent change; 95%CI, 95% confidence interval; *p < 0.05.

The estimated breast cancer screening coverage for women ≥70 years of age within the public healthcare network for the 2013–2019 period fell from 13.3 to 10.8%. Regression analysis showed a significant reduction in screening for both groups, with that reduction being more pronounced in the ≥70 years group, with an APC of-3.51 (95%CI: −4.0 to –3.0; *p* < 0.001) compared to the 50–69 years group, with an APC of –1.78 (95%CI: −3.4 to-0.1; *p* = 0.040; Figure 1).

According to the regression analysis, for the ≥70 years group there was a trend towards a reduction in all the regions over the entire period analyzed: Midwest: APC-5.88 (95%CI: −11.1 to –0.3; *p* = 0.041), North: APC –4.90 (95%CI: −9.0 to –0.6; *p* = 0.003), Northeast: APC-5.03 (95%CI: −7.2 to –2.8, *p* = 0.002), Southeast: APC –3.29 (95%CI: −4.3 to –2.2; *p* = 0.001) and South: APC –1.52 (95%CI: −2.0 to –0.1; *p* = 0.039), as shown in Figure 1. For the 50–69 years group, there was a trend toward stability in the Midwest, North, and Northeast, with a trend towards a reduction in the other regions: Southeast: APC-1.86 (95%CI: −3.6 to –0.1, *p* = 0.042) and South: APC –1.27 (95%CI: −2.3 to –0.2, *p* = 0.026; Figure 1).

Table 3 shows the distribution of clinical staging for the two age groups according to the geographical regions. Considering the country as a whole, there was a greater occurrence of stages III and IV in the ≥70 group (44.3%) compared to the women of 50–69 years of age (40.8%; *p* < 0.001).

**Table 3:** Characterization of staging between 2013 and 2019 according to age group for Brazil and its geographical regions.

Staging/Regions	Age group		
	50–69 years *n* (%)	≥70 years *n* (%)	Value of p*
Brazil			
I and II	48,567 (59.2)	15,387 (55.7)	**0.001**
III and IV	33,440 (40.8)	12,258 (44.3)	
Midwest			
I and II	2,378 (54.2)	618 (53.5)	0.642
III and IV	2008 (45.8)	538 (46.5)	
Northeast			
I and II	11,387 (56.9)	3,625 (55.0)	**0.007**
III and IV	8,631 (43.1)	2,969 (45.0)	
North			
I and II	1,310 (55.4)	304 (49.6)	**0.010**
III and IV	1,053 (44.6)	309 (50.4)	
Southeast			
I and II	22,426 (60.2)	7,332 (56.0)	**0.001**
III and IV	14,847 (39.8)	5,759 (44.0)	
South			
I and II	11,066 (61.6)	3,508 (56.7)	**0.001**
III and IV	6,901 (38.4)	2,683 (43.3)	

Pearson’s chi-square, absolute frequency (relative frequency), *p< 0.05.The meaning of the bold values provided in table is the geographical regions of Brazil.

**Figure 2:**
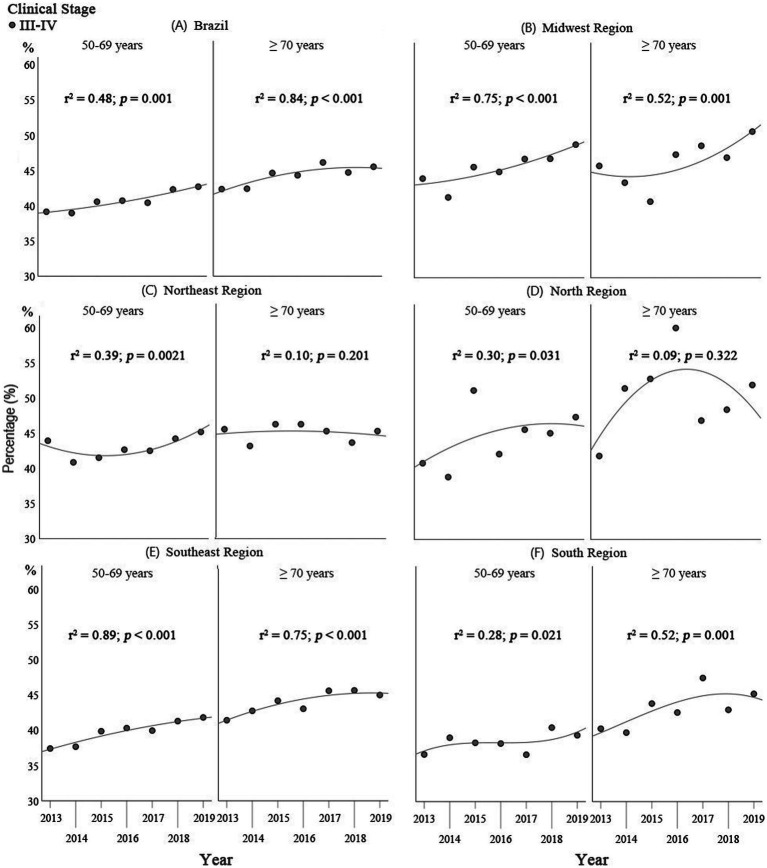
Comparison of the results of the Poisson regression analysis on staging in Brazil as a whole and for the geographical regions: **(A)** Brazil; **(B)** Midwest; **(C)** North; **(D)** Northeast**; (E)** Southeast; and **(F)** South, between women ≥70 years of age and those of 50–69 years of age for the 2013–2019 period.

In the regression analysis on staging, for the group of women ≥70 years of age there was a trend towards an increase in stages III and IV breast cancer for Brazil as a whole and for the Midwest, Southeast and South (Figure 2), with coefficients of determination (R2) of 0.84 (*p* < 0.001), 0.52 (*p* < 0.001), 0.75 (*p* < 0.001), and 0.52 (*p* = 0.001), respectively. In the Northeast and North, there was no defined trend with respect to early and advanced cancer stages, with R2 of 0.10 (*p* < 0.201) and 0.09 (*p* = 0.322), respectively, over the study period (Figure 2).

For the group of women of 50–69 years of age, there was a trend towards an increase in the proportion of stages III and IV in Brazil as a whole and in the Midwest and Southeast (Figure 2), with coefficients of determination (*R*^2^) of 0.48 (*p* = 0.001), 0.75 (*p* < 0.001), and 0.89 (*p* < 0.001), respectively. In the Northeast, North and South, there was no defined trend towards early or late breast cancer staging, with *R*^2^ of 0.38 (*p* = 0.021), 0.30 (*p* = 0.031), and 0.28 (*p* = 0.021), respectively, over the study period (Figure 2).

## 4. Discussion

### 4.1. Profile of the older adults female population in Brazil

In Brazil, the female population has benefited from an overall reduction in mortality rates and a consequent increase in the likelihood of survival until 60–80 years of age. The group of Brazilian women of 70 years of age or more increased by 44% between 2010 and 2020. For the next 20 years, an increase of 113% is expected in this segment of the population, reaching a total of 16,746,544 women (35).

A large proportion of the older adults Brazilian population remains independent (36, 37), with around half having fewer than two diseases (37). This profile supports the need of an individualized analysis of older adults women with respect to maintaining breast cancer screening.

### 4.2. Breast cancer screening, clinical staging and mortality rate

To the best of our knowledge, this is the first study conducted in Brazil to evaluate breast cancer screening rates and breast cancer staging in women ≥70 years of age within the public healthcare system. As shown, between 2013 and 2019 there was a trend towards a reduction in the screening rate and an increase in the incidence of advanced stages of breast cancer at diagnosis. These findings are in dissonance with the increase in the older adults female segment of the Brazilian population and may represent one of the causes of the increase in mortality from breast cancer in this age group.

In the present study, the coefficient of determination was found to be higher in the over-70s (*R*^2^ 0.84, *p* < 0.001) compared to the 50–69-year old group, the group for which the Ministry of Health recommends screening (*R*^2^ 0.48, *p* < 0.001). This reflects a greater trend towards an increase in advanced stages of the disease in the group of older adults women ≥70 years of age. This finding could be a consequence of delayed diagnosis of the disease, bearing in mind that tumor growth tends to be more indolent in this age group (23). In this respect, the current cut-off limit of 69 years of age for breast cancer screening can be included as one of the explanations for the occurrence of advanced tumors in the older adults population (23).

Breast cancer screening coverage provided under the Brazilian National Public Health Service, to women aged 50 to 69 years of age (many currently belong to the ≥70 years of age group) ranged from 14.4 to 24.2% during 2008–2017. Evaluation of the temporal changes in breast cancer screening coverage showed an initial increase, followed by stability (32). In the present study, the results for the years after this period in this age group were analyzed and showed a trend towards a reduction in mammographic coverage in the country as a whole. These data suggest that public policies were insufficient to ensure organized screening.

Moreover, there is a reduction in breast cancer screening in the over-seventies in Brazil leading to the assumption that women screened between 50 and 69 years of age did not continue screening after ≥70 years of age, which may partially explain the increase in prevalence of advanced disease stages at diagnosis in this age group. Similar findings could be expected in other LMIC.

This finding highlights the urgent need to review public policies for this group in order to foment diagnosis at an earlier stage and, consequently, implement timely treatment. Public healthcare in Brazil has failed to accompany the international trend of increasing breast cancer screening to include women ≥70 years of age whose functional status, comorbidities and life expectancy permit this indication. The Ministry of Health recommendations (19) contraindicate screening for this age group, rejecting a multidimensional evaluation of the older adults woman, a fact that justifies the poor breast cancer screening rate highlighted in the present study.

Clinically, older women tend to present with larger tumors and lymph node involvement at diagnosis, probably due to delayed diagnosis (25). In Canada, Germany and Norway, stages I and II are more common than stages III and IV (20–22). Reports from other countries have shown varying results with respect to clinical staging, including an increase in the incidence of stage II and III disease in women over 80 years of age (23) and an increase in the prevalence of stage IV (24).

Regarding molecular subtype, specific data in the older adults population also appear to be limited. Luminal A breast cancer is the most common subtype in women of all races and ethnicities worldwide (38), followed by luminal B HER2-negative, HER2-positive, and triple-negative breast cancer (39). In Brazil, the most common breast cancer subtype was luminal A (48.0%), followed by luminal B-HER2 positive-like (17.0%) and triple-negative (15.6%) (40). Given the similarity of the data in Brazil and worldwide, we do not believe that molecular subtypes affect the staging results found in the present study.

The higher mortality rate in this group is probably due to the lack of breast cancer screening, the more advanced stages of the disease at diagnosis, under-treatment and the presence of multiple comorbidities (25).

### 4.3. Need for evaluation that goes beyond chronological age

Age alone should not define any aspect of the management of older adults women with breast cancer, as chronological age is unable to predict an individual’s physiological decline, since comorbidities and other determinants all play a role in the body’s aging process (12, 13, 41). All decisions should take the following factors into consideration: physiological age, estimated life expectancy, risks, benefits, tolerance to treatment, the patient’s preference and potential barriers to treatment (4, 42–45).

Mortality indexes that use age, comorbidities and functional status to predict life expectancy over the long term have been used in other countries to support decisions regarding when to cease screening (6, 8, 44). Identifying women with sufficient life expectancy to benefit from screening would minimize the harm associated with false-positive results and over-diagnosis in women who will not live long enough to obtain benefit (13, 16).

The aging of the Brazilian population has triggered demands to change public policies, with different services for this group being required (46, 47). In view of the specificity and heterogeneity involved in the aging process, a multidimensional evaluation is required that encompasses functionality, fragility risk, degree of dependence, cognitive ability (46, 48), and the social and family context (48) whenever elaborating a treatment plan. Indeed, decisions regarding breast cancer screening and treatment cannot be based on age alone (49).

The study limitations include its retrospective design and the use of secondary data that limit the analysis of other important variables in the context of the older adults population. Nevertheless, this large and representative population sample increases the robustness and internal validation of the study. Furthermore, the quality of the data obtained from DATASUS, whose records have functioned autonomously and independently for more than 20 years, merits particular mention. Finally, we believe that the data from the present study highlight a need to reformulate actions of screening, diagnosis and treatment in women ≥70 years of age of age in Brazil, and may serve as an alert to public managers in other LMIC.

## 5. Conclusion

In view of the aging Brazilian population and the heterogeneity of the functional and cognitive status of older adults women, breast cancer screening in the group of women ≥70 years of age in the Brazilian National Health Service merits further debate within the realm of implanting public policies. Equity should be sought between the different regions of the country so that all older adults women for whom it is recommended have the opportunity to undergo breast cancer screening, identifying means of meeting this growing demand with quality and resoluteness.

Basing the decision to cease breast cancer screening on chronological age is insufficient to deal with the multi-dimensionality of aging. The perception of the aging process needs to be broadened, using measures to evaluate functional/cognitive status, comorbidity index and the estimated number of years of breast cancer-specific survival.

## Data availability statement

The raw data supporting the conclusions of this article will be made available by the authors, without undue reservation.

## Ethics statement

The internal review board of the Federal University of Goiás Teaching Hospital approved the study protocol under reference CAAE: 56747022.0.0000.5078. Written informed consent for participation was not required for this study in accordance with the national legislation and the institutional requirements.

## Author contributions

AR and RF-J: conceptualization and methodology. AR: validation, investigation, and writing – original draft preparation. AR and GF: formal analysis. AR and LS: resources. AR, RF-J, LS, and GF: writing – review and editing, visualization, and supervision. All authors have read and agreed to the published version of the manuscript.

## Conflict of interest

The authors declare that the research was conducted in the absence of any commercial or financial relationships that could be construed as a potential conflict of interest.

## Publisher’s note

All claims expressed in this article are solely those of the authors and do not necessarily represent those of their affiliated organizations, or those of the publisher, the editors and the reviewers. Any product that may be evaluated in this article, or claim that may be made by its manufacturer, is not guaranteed or endorsed by the publisher.

## References

[1] Sung H Ferlay J Siegel RL Laversanne M Soerjomataram I Jemal A. Global Cancer Statistics 2020: GLOBOCAN estimates of incidence and mortality worldwide for 36 cancers in 185 countries. CA Cancer J Clin. (2021) 71:209–49. doi: 10.3322/caac.21660, PMID: 33538338

[2] IARC. Brazil – source: Globocan 2020. Incidence, mortality and prevalence by cancer site. Global Cancer Observatory. (2021). Available at: https://gco.iarc.fr/today/data/factsheets/populations/76-brazil-fact-sheets.pdf (Accessed March 31, 2023).

[3] Santos MO Lima FCS Martins LFL Oliveira JFP Almeida LM Cancela MC. Estimated cancer incidence in Brazil, 2023-2025. Rev. Bras. Cancerol. (2023) 69:e-213700. doi: 10.32635/2176-9745.RBC.2023v69n1.3700

[4] Gu YF Lin FP Epstein RJ. How aging of the global population is changing oncology. Ecancermedicalscience. (2021) 15:ed119. doi: 10.3332/ecancer.2021.ed119, PMID: 35211208 PMC8816510

[5] Lee CS Moy L Joe BN Sickles EA Niell BL. Screening for breast cancer in women age 75 years and older. AJR Am J Roentgenol. (2018) 210:256–63. doi: 10.2214/AJR.17.18705 29112471

[6] Oeffinger KC Fontham ET Etzioni R Herzig A Michaelson JS Shih YC. American Cancer Society. Breast cancer screening for women at average risk: 2015 Guideline update from the American Cancer Society. JAMA. 314:1599–614. doi: 10.1001/jama.2015.12783 26501536 PMC4831582

[7] Urban LABD Chala LF Bauab SP Schaefer MB Santos RP Maranhão NMA. Breast cancer screening: updated recommendations of the Brazilian College of Radiology and Diagnostic Imaging, Brazilian breast disease society, and Brazilian Federation of Gynecological and Obstetrical Associations. Radiol Bras. (2017) 50:244–9. doi: 10.1055/s-0037-1606348, PMID: 28894332 PMC5586515

[8] Kotwal AA Walter LC. Cancer screening among older adults: a geriatrician’s perspective on breast, cervical, colon, prostate, and lung cancer screening. Curr Oncol Rep. (2020) 22:108. doi: 10.1007/s11912-020-00968-x, PMID: 32803486 PMC8191500

[9] Silva GAE Souza-Júnior PRB Damacena GN Szwarcwald CL. Early detection of breast cancer in Brazil: data from the National Health Survey, 2013. Rev Saude Publica. (2017) 51:14S–8S. doi: 10.1590/S1518-8787.2017051000191, PMID: 28591356 PMC5676402

[10] Walter LC Schonberg MA. Screening mammography in older women: a review. JAMA. (2014) 311:1336–47. doi: 10.1001/jama.2014.2834, PMID: 24691609 PMC4391705

[11] Lee CS Sengupta D Bhargavan-Chatfield M Sickles EA Burnside ES Zuley ML. Association of patient age with outcomes of current-era, large-scale screening mammography: analysis of data from the national mammography database. JAMA Oncol. (2017) 3:1134–6. doi: 10.1001/jamaoncol.2017.0482, PMID: 28426842 PMC5793704

[12] Monticciolo DL. Current guidelines and gaps in breast cancer screening. J Am Coll Radiol. (2020) 17:1269–75. doi: 10.1016/j.jacr.2020.05.002, PMID: 32473894

[13] Kotwal AA Schonberg MA. Cancer screening in the elderly: a review of breast, colorectal, lung, and prostate cancer screening. Cancer J. (2017) 23:246–53. doi: 10.1097/PPO.0000000000000274, PMID: 28731949 PMC5608027

[14] Mandelblatt JS Stout NK Schechter CB van den Broek JJ Miglioretti DL Krapcho M. Collaborative modeling of the benefits and harms associated with different U.S. breast cancer screening strategies. Ann Intern Med. (2016) 164:215–25. doi: 10.7326/M15-1536, PMID: 26756606 PMC5079106

[15] International Agency for Research on Cancer (IARC). Breast Cancer screening, 2nd IARC handbooks of Cancer prevention: Lyon, France, (2016) 15

[16] Braithwaite D Demb J Henderson LM. Optimal breast cancer screening strategies for older women: current perspectives. Clin Interv Aging. (2016) 11:111–25. doi: 10.2147/CIA.S65304, PMID: 26893548 PMC4745843

[17] Monticciolo DL Malak SF Friedewald SM Eby PR Newell MS Moy L. Breast cancer screening recommendations inclusive of all women at average risk: update from the ACR and Society of Breast Imaging. J Am Coll Radiol. (2021) 18:1280–8. doi: 10.1016/j.jacr.2021.04.021, PMID: 34154984

[18] American Geriatrics Society. Ten things clinicians and patients should question. Last reviewed (2021) Available at: https://www.choosingwisely.org/wp-content/uploads/2015/02/AGS-Choosing-Wisely-List.pdf.

[19] Migowski A Silva GAE Dias MBK Diz MDPE Santna DR Nadanovsky P. Guidelines for early detection of breast cancer in Brazil. II – new national recommendations, main evidence, and controversies. Cad Saude Publica. (2018) 34:e00074817. doi: 10.1590/0102-311X00074817 29947654

[20] Bryan S Masoud H Weir HK Woods R Lockwood G Smith L. Cancer in Canada: stage at diagnosis. Health Rep. (2018) 29:21–5. PMID: 30566206

[21] Chatzidaki P Mellos C Briese V Mylonas I. Does primary breast cancer in older women (≥80 years) have unfavorable histological characteristics? Arch Gynecol Obstet. (2011) 284:705–12. doi: 10.1007/s00404-010-1697-5, PMID: 20949358

[22] Johansson ALV Trewin CB Hjerkind KV Ellingjord-Dale M Johannesen TB Ursin G. Breast cancer-specific survival by clinical subtype after 7 years follow-up of young and elderly women in a nationwide cohort. Int J Cancer. (2019) 144:1251–61. doi: 10.1002/ijc.31950, PMID: 30367449

[23] Bertolo A Rosso C Voutsadakis IA. Breast cancer in patients 80 years-old and older. Eur J Breast Health. (2020) 16:208–12. doi: 10.5152/ejbh.2020.5659, PMID: 32656522 PMC7337909

[24] Rottenberg Y Naeim A Uziely B Peretz T Jacobs JM. Breast cancer among older women: the influence of age and cancer stage on survival. Arch Gerontol Geriatr. (2018) 76:60–4. doi: 10.1016/j.archger.2018.02.004 29459246

[25] Desai P Aggarwal A. Breast cancer in women over 65 years - a review of screening and treatment options. Clin Geriatr Med. (2021) 37:611–23. doi: 10.1016/j.cger.2021.05.007 34600726

[26] DATASUS. SUS-SIASUS outpatient data system. Department of Information Technology of SUS-DATASUS. (2016). Available at: http://tabnet.datasus.gov.br/cgi/deftohtm.exe?sia/cnv/qbuf.def.

[27] DATASUS. Brazil panel-oncology. Cancer treatment monitoring panel (2022). Available at: http://tabnet.datasus.gov.br/cgi/dhdat.exe?PAINEL_ONCO/PAINEL_ONCOLOGIABR.def (Accessed April 17, 2022).

[28] IBGE. Brazilian Institute of Geography and Statistics. Projections for the population of Brazil and its states by sex and age: 2010-2060. (2018). Available at: https://www.ibge.gov.br/estatisticas/sociais/populacao/9109-projecao-da-populacao.html?=&t=resultados (Accessed March 19, 2022).

[29] ANS. National Agency for supplemental health. Health insurance coverage rate. (2022) Available at: http://www.ans.gov.br/anstabnet/cgi-bin/tabnet?dados/tabnet_tx.def (Accessed April 17, 2022).

[30] INCA. Online atlas of cancer mortality. (2014) Available at: https://mortalidade.inca.gov.br/MortalidadeWeb/pages/Modelo10/consultar.xhtml (Accessed April 5, 2022).

[31] INCA. Technical parameters for breast cancer screening. Rio de Janeiro: INCA. (2021) Available at: http://controlecancer.bvs.br/ (Accessed April 1, 2023).

[32] Rodrigues DCN Freitas-Junior R Rahal RMS da Silveira Corrêa R Gouveia PA Peixoto JE. Temporal changes in breast cancer screening coverage provided under the Brazilian National Health Service between 2008 and 2017. BMC Public Health. (2019) 19:959. doi: 10.1186/s12889-019-7278-z, PMID: 31319826 PMC6637648

[33] National Cancer Institute. Joinpoint regression program. Statistical methodology and applications branch, surveillance research program, National Cancer Institute. (2022). Available at: https://surveillance.cancer.gov/joinpoint/ (Accessed February 12, 2022).

[34] International Business Machines Corporation. SPSS statistics for windows. Armonk, NY: IBM (2019).

[35] IBGE. Brazilian Institute of Geography and Statistics. Complete mortality table for Brazil - 2019: brief analysis of the evolution of mortality in Brazil. (2020). Available at: https://agenciadenoticias.ibge.gov.br/media/com_mediaibge/arquivos/65c3023462edaabf0d7318c1a0f80ca4.pdf (Accessed April 01, 2023).

[36] BRASIL. Ministry of Health. Guidelines for the care of the elderly in the SUS: a proposal for a comprehensive care model. (2014). Available at: https://bvsms.saude.gov.br/bvs/publicacoes/diretrizes_cuidado_pessoa_idosa_sus.pdf (Accessed April 01, 2023).

[37] IBGE. Perception of health status, lifestyles, chronic diseases and oral health: Brazil and major regions. Rio de Janeiro: IBGE (2020). 113 p.

[38] Grabinski VF Brawley OW. Disparities in breast cancer. Obstet Gynecol Clin N Am. (2022) 49:149–65. doi: 10.1016/j.ogc.2021.11.010 35168767

[39] Harbeck N Penault-Llorca F Cortes J Gnant M Houssami N Poortmans P. Breast cancer. Nat Rev Dis Primers. (2019) 5:66. doi: 10.1038/s41572-019-0111-2 31548545

[40] Rosa DD Bines J Werutsky G Barrios CH Cronemberger E Queiroz GS. The impact of sociodemographic factors and health insurance coverage in the diagnosis and clinicopathological characteristics of breast cancer in Brazil: AMAZONA III study (GBECAM 0115). Breast Cancer Res Treat. (2020) 183:749–57. doi: 10.1007/s10549-020-05831-y, PMID: 32728860

[41] Silva LCR Amorim WC Castilho MS Guimarães RC Paixão TP Pirfo CBL. Breast cancer in women over 70 years of age: guidelines for diagnosis and treatment. Rev Med Minas Gerais. (2013) 23:105–12. doi: 10.5935/2238-3182.20130016

[42] Biganzoli L Battisti NML Wildiers H McCartney A Colloca G Kunkler IH. Updated recommendations regarding the management of older patients with breast cancer: a joint paper from the European Society of Breast Cancer Specialists (EUSOMA) and the International Society of Geriatric Oncology (SIOG). Lancet Oncol. (2021) 22:e327–40. doi: 10.1016/S1470-2045(20)30741-5, PMID: 34000244

[43] Cadet T Aliberti G Karamourtopoulos M Jacobson A Gilliam EA Primeau S. Evaluation of a mammography decision aid for women 75 and older at risk for lower health literacy in a pretest-posttest trial. Patient Educ Couns. (2021) 104:2344–50. doi: 10.1016/j.pec.2021.02.020, PMID: 33637391 PMC8364563

[44] Parks RM Cheung KL. Personalising care in the older woman with primary breast cancer. Ann Acad Med Singap. (2019) 48:370–5. doi: 10.47102/annals-acadmedsg.V48N11p370 31960017

[45] Schrager S Ovsepyan V Burnside E. Breast cancer screening in older women: the importance of shared decision making. J Am Board Fam Med. (2020) 33:473–80. doi: 10.3122/jabfm.2020.03.190380, PMID: 32430383 PMC7822071

[46] Vega E Morsch P. The decade of healthy aging (2021-2030) in the region of the Americas. Mais60-Estudos sobre Envelhecimento. (2021). Available at: https://www.sescsp.org.br/ed-80-a-decada-do-envelhecimento-saudavel-2021-2030-na-regiao-das-americas/ (Accessed April 1, 2023).

[47] BRASIL. Technical guidelines for the implementation of the line of Care for Comprehensive Health Care for the elderly in the unified health system – SUS. Ministry of Health, Department of Health Care, Department of Programmatic and Strategic Actions. Brasília: Ministério da Saúde. (2018) Available at: http://bvsms.saude.gov.br/bvs/publicacoes/linha_cuidado_atencao_pessoa_idosa.pdf (Accessed April 01, 2023).

[48] BRASIL Ministry of Health, Sociedade Beneficente Israelita Brasileira Albert Einsten. Technical note for the organization of the health care network with a focus on primary health care and specialized outpatient care - health for the elderly. (2019) Available at: https://atencaobasica.saude.rs.gov.br/upload/arquivos/202001/03091212-nt-saude-do-idoso-planificasus.pdf (Accessed April 17, 2022).

[49] Shachar SS Hurria A Muss HB. Breast cancer in women older than 80 years. J Oncol Pract. (2016) 12:123–32. doi: 10.1200/JOP.2015.010207 26869650

